# Evaluation of the Relationship between Nutritional Status of COVID-19 Patients Admitted to the ICU and Patients' Prognosis: A Cohort Study

**DOI:** 10.1155/2022/5016649

**Published:** 2022-07-18

**Authors:** Parsa Mohammadi, Hesam Aldin Varpaei, Alireza Khafaee pour khamseh, Mostafa Mohammadi, Mojgan Rahimi, Amirhossein Orandi

**Affiliations:** ^1^Faculty of Medicine, Tehran University of Medical Sciences, Tehran, Iran; ^2^Department of Surgical Nursing, Faculty of Nursing, Near East University, Nicosia, Cyprus; ^3^Faculty of Medicine, Islamic Azad University Tehran Medical Sciences, Tehran, Iran; ^4^Department of Anesthesiology and Critical Care, Tehran University of Medical Sciences, Tehran, Iran

## Abstract

**Background:**

Malnutrition in COVID-19 critically ill patients can lead to poor prognosis. This study aimed to evaluate the association between nutritional status (or risk) and the prognosis of critically ill COVID-19 patients. In this study, prognosis is the primary outcome of “hospital mortality” patients. The second outcome is defined as “need for mechanical ventilation.”

**Methods and Materials:**

In this single-center prospective cohort study, 110 patients admitted to the Intensive Care Unit of Imam Khomeini Hospital Complex (Tehran, Iran) between April and September 2021 were enrolled. Participants formed a consecutive sample. MNA-SF, NRS-2002, mNUTRIC, and PNI scores were used to evaluate nutritional assessment. Patients' lab results and pulse oximetric saturation SpO_2_/FiO_2_ (SF) ratio at the time of intensive care unit (ICU) admission were collected. Patients were screened for nutritional status and categorized into two groups, patients at nutritional risk and nonrisk.

**Results:**

Sixty-five (59.1%) of all patients were men. The overall range of age was 52 ± 15. Thirty-six (32.7%) of patients were obese (BMI ≥ 30). The hospital mortality rate was 59.1% (*n* = 65). According to the different criteria, malnutrition rate was 67.3% (*n* = 74) (NRS), 28.2% (*n* = 31) (MNA), 34.5% (*n* = 38) (mNUTRIC), and 58.2% (*n* = 64) (PNI). There was a statistically significant association between chronic kidney disease (CKD) and mNUTRIC risk (OR = 13.5, 95% CI (1.89–16.05), *P*=0.002), diabetes mellitus (DM) and MNA risk (OR = 2.82, 95% CI (1.01–7.83), *P*=0.041), hypertension (HTN) and MNA risk (OR = 5.63, 95% CI (2.26–14.05), *P* < 0.001), and malignancy and mNUTRIC risk (*P*=0.048). The nutritional risk (all tools) significantly increased the odds of in-hospital death and need for mechanical ventilation. The length of stay was not significantly different in malnourished patients.

**Conclusion:**

In the critical care setting of COVID-19 patients, malnutrition is prevalent. Malnutrition (nutritional risk) is associated with an increased risk of need for mechanical ventilation and in-hospital mortality. Patients with a history of HTN, CKD, DM, and cancer are more likely to be at nutritional risk at the time of ICU admission.

## 1. Introduction

Outbreaks of severe acute respiratory syndrome coronavirus 2 (SARS-CoV-2) caused by the new coronavirus (2019-nCoV) have spread rapidly throughout the world. The clinical spectrum of coronavirus disease (COVID-19) varies from asymptomatic infection to severe pneumonia [1]. Because COVID-19 is an acute inflammatory phase (manifested as an unavoidable process in the form of fever, loss of appetite, and weight loss), patients must be able to withstand the inflammatory cascade resulting from those cytokine storms [2]. Given that the course of COVID-19 is likened to a waterfall, it seems that having a normal nutritional status can play a role in preventing the patient from falling into the abyss of the waterfall. Also, it is suggested that nutritional assessment should be done at the time of hospitalization [3].

Nutritional status plays a crucial role in the onset of severe disease and prognosis of SARS-CoV-2. Poor nutritional status can affect the immune system and inflammatory process and cause challenges for health professionals in the treatment sector [4]. Nutritional risk is predicted by some international methods like Nutritional Risk Screening 2002 (NRS-2002), Mini Nutritional Assessment-Short Form (MNA-SF), and modified Nutrition Risk in the Critically III (mNUTRIC) score. These are some useful and simple ways of screening for patients who are admitted to a ward or intensive care unit (ICU) [5]. It seems that there is an association between serum albumin, body mass index (BMI), and malnutrition. BMI ≥ 30 kg/m^2^ patients appear to have a poor prognosis (ARDS, severe COVID-19, need of mechanical ventilation, and hospital admission), and higher BMI is not associated with ICU admission but may prolong the length of stay (LOS) at the hospital [6, 7]. A lower level of serum albumin was seen in critically ill COVID-19 patients with poor prognosis [8]. It has been claimed that low serum albumin is associated with nutritional risk in ESRD [9], atrial fibrillation [10], and critically ill COVID-19 patients' disease severity and outcome [8].

Some studies have shown that a high score of mNUTRIC (≥5 points) in ICU patients can increase the mortality rate [11]. Another study mentioned that NRS-2002 ≥ 3 is a risk factor for mortality in COVID-19 patients [12]. Weixing Wang et al. [13] evaluated NRS-2002 and MNA-SF in well-nourished and nutritional risk groups of COVID-19 patients and suggested that there is a significant difference in LOS, hospital expenses, and severity of disease between them. According to one of the findings of a study conducted in Portugal [14], high mNUTRIC (≥5 points) is associated with need of mechanical ventilation in ICU patients. In previous studies, the relationship between nutritional risk in critically ill and noncritically ill patients has been investigated. Mostly, they used one or two nutrition assessment tools. The present study mainly focuses on ICU patients with four different nutrition assessment tools to determine nutritional risk and associated factors. Therefore, we aimed to evaluate the association between nutritional status (or risk) and the prognosis of critically ill COVID-19 patients. In this study, prognosis is the primary outcome of “hospital mortality” patients. The second outcome defined as “need for mechanical ventilation.”

## 2. Methods and Materials

In this single-center prospective cohort study, we enrolled 110 patients admitted to the intensive care units of Imam Khomeini Hospital Complex (IKHC) between April and September, 2021 (participants formed a consecutive sample). IKHC is a government and educational hospital (1000 beds and 4 ICU) in Tehran (capital of Iran), which is managed by Tehran University of Medical Sciences. Data were collected from General ICU of IKHC. This study was carried out in conformity with the World Medical Association's Declaration of Helsinki after receiving clearance from the University Ethics Committee (IR.TUMS.IKHC.REC.1400.229). The inclusion criteria were SARS-CoV-2 infection verified by real-time polymerase chain reaction (PCR) and age between 18 and 80 years. Patients admitted with multiorgan failures and intubated patients admitted to the ICU were excluded.

Patients' lab results and pulse oximetric saturation SpO_2_/FiO_2_ (SF) ratio at the time of ICU admission were collected. Also, standardized scores including Acute Physiologic Assessment and Chronic Health Evaluation II (APACHE II) at admission time and Sequential Organ Failure Assessment (SOFA) within the first 3 days of ICU stay were collected. Four tools were utilized to assess patients' nutritional status: Mini Nutritional Assessment-Short Form (MNA-SF), Nutritional Risk Screening 2002 (NRS-2002), modified NUTRIC score (mNUTRIC), and Prognostic Nutritional Index (PNI).

NRS-200 questionnaire [15]: the NRS-2002 questionnaire includes initial and final evaluation. Parameters of weight loss, percentage of reduced food intake, body mass index, disease severity, disease type, and age are considered. A score of less than 3 is normal, and more than or equal to 3 is considered a nutritional risk.

MNA-SF score (Mini Nutritional Assessment-Short Form) [16]: this questionnaire includes nutritional status assessment parameters including six sections; these parameters are loss of appetite, recent weight loss, mobility, recent acute illness, dementia or depression, and body mass index or measure the leg circumference. This questionnaire has 14 scores, the numerical range of 12–14 indicates normal nutritional status, 8–11 indicates at risk of malnutrition, and less than 7 indicates malnutrition.

NUTRIC score [17] is a tool for assessing the nutritional risk of patients in critical situations. It evaluates six variables, which are age, APACHE II, SOFA, number of comorbidities, number of days hospitalized until admission to the intensive care unit, and interleukin-6 measurement for evaluation. There are scores for this instrument with and without interleukin-6, which in both cases is a valid tool for assessing the nutritional risk in patients in critical conditions. In the form without interleukin-6, the NUTRIC score is from 0 to 9 (called mNUTRIC). A score of 0–4 indicates a low risk of nutritional index and a score of 5–9 indicates a high risk of nutritional index in patients admitted to intensive care units and the need to start severe nutritional interventions in these patients.

PNI: this nutritional status assessment index [18] is evaluated according to the following formula:

(1) $$\begin{array}{c} 10\times \text{serum albu }in\left(\frac{g}{\text{d}l}\right)+0.005\times \text{total lymphocyte count}\left(\text{per mm}\right). \end{array}$$

### 2.1. Nutritional Risk Assessment

Patients were screened for nutritional status and categorized into two groups, nutritional risk and nonrisk. According to nutrition assessment tools, nutritional risk is considered as NRS-2002 ≥ 3, mNUTRIC ≥ 5, MNA < 12, and PNI < 35.

### 2.2. Outcome

Patients were followed up until the final outcome (discharge or expiration) and whether they needed mechanical ventilation during the ICU stay or not. In this study, prognosis is the primary outcome of “mortality” patients. The second outcome is defined as “need for mechanical ventilation.”

### 2.3. Statistical Analysis

**Figure 1:**
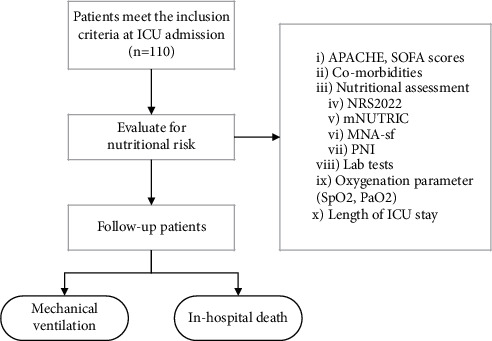
Flowchart of patients included in the study.

The threshold for statistical significance was set at a two-sided *P* value of 0.05. IBM SPSS v26 was used to conduct all of the analyses. Demographic and medical data that met the normal distribution criteria were represented as mean ± standard deviation. The median was used to represent data having a skewed distribution (IQR). Frequency rates and percentages were used to describe categorical variables. The Kolmogorov–Smirnov test was used to evaluate the normality distribution. The differences between discharged and expired patients were analyzed using a two-sample *t*-test or a Mann–Whitney *U*-test depending on whether the data were normal or skewed, and a chi-squared test for categorical variables. A logistic regression was performed to ascertain the effects of nutritional risk (according to different scores) on the likelihood of a patient's survival and need for mechanical ventilation. The connection between dietary and metabolic parameters and the risk of mortality in the hospital was investigated using logistic regression. A receiver operating characteristic curve (ROC) was used to test the accuracy of MNA-SF, NRS-2002, mNUTRIC, and PNI to predict hospital mortality, as shown in Figure 1.

## 3. Results

Sixty-five (59.1%) of the all patients were men. The overall range of age was 52 ± 15. Thirty-six (32.7%) of patients were obese (BMI ≥ 30). Mortality rate was 59.1%(*n* = 65). The prevalence of underlying diseases was hypertension (HTN) 28.2%, diabetes (DM) 17.3%, heart disease 18.2%, chronic kidney disease (CKD) 7.2%, neurological disorders 3.6%, hypothyroidism 3.6%, liver disease 1.8%, and asthma 0.9%. Sixty-eight (61.8%) patients need mechanical ventilation during ICU stay. In the case of nutritional risk assessment, according to the criteria, malnutrition rate was 67.3% (NRS), 28.2% (MNA), 34.5% (mNUTRIC), and 58.2% (PNI). There was a statistically significant association between CKD and mNUTRIC risk (OR = 13.5, 95% CI (1.89–16.05), *P*=0.002), DM and MNA risk (OR = 2.82, 95% CI (1.01–7.83), *P*=0.041), HTN and MNA risk (OR = 5.63, 95% CI (2.26–14.05), *P* < 0.001), and malignancy and mNUTRIC risk (OR = 8.83, *P*=0.048).

According to BMI categories (obese and nonobese), there were some associations. Obese patients were more likely to be exposed (higher odds ratio) to the nutritional risk according to PNI (OR = 3, *P*=0.01), NRS (OR = 2.6, *P*=0.03), and mNUTRIC (OR = 2.7, *P*=0.01). A Mann–Whitney *U*-test was performed to compare the mean ranks of variables between the expired and discharged patients (Table 1).

Nonsurvival patients had higher BMI, APACHE, and SOFA scores significantly (*P* < 0.005). Survival patients (discharged) had higher serum albumin, MNA-SF, and PNI. But they had lower NRS and mNUTRIC scores in comparison to the expired patients. The WBC, neutrophil count, and N/L ratio of nonsurvival patients were significantly higher than discharged patients. However, they had lower lymphocyte count and SF ratio significantly (*P* < 0.005). Length of ICU stay did not show significant differences (*P* < 0.05). Although there was no significant difference in ESR levels between the two groups of patients, three inflammatory biomarkers, CRP, LDH, and ferritin, were significantly higher in the expired patients (*P* < 0.05).

**Figure 2:**
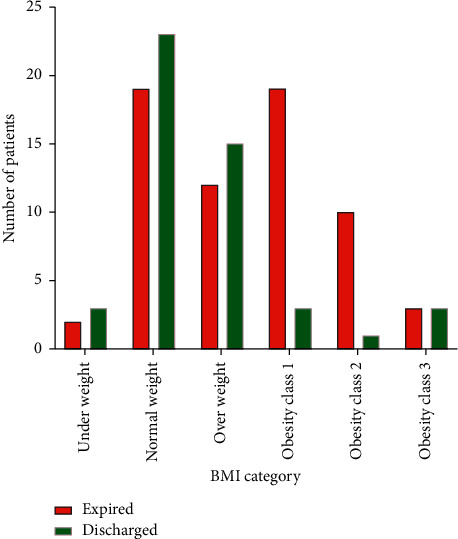
Patients' in-hospital mortality according to BMI categories.

The chi-square test was performed to assess the supposed association between nutritional risk and discharge from the hospital and need for mechanical ventilation (Tables 2 and 3). In-hospital mortality was significantly associated with BMI categories (*P*=0.001). Obese patients had higher mortality rate in comparison to underweight, overweight, and normal weight patients (Figure 2).

Obesity (BMI > 30) is associated with a decrease in the odds of discharge from the hospital for patients admitted to the ICU (OR = 0.10, 95% CI (0.032–0.311), *P* < 0.001). Also, obesity increased the odds of need for mechanical ventilation significantly (OR = 8.4, 95% CI (2.714–26.272), *P* < 0.001). It is evident that obese patients in the ICU are 8.4 times more likely to need mechanical ventilation in comparison to nonobese patients.

The nutritional risk (all tools) significantly increased the odds of in-hospital death and also need for mechanical ventilation (Table 3). It is vivid that nutritional risk can decrease the probability of discharge from the hospital (survival).

A logistic regression was performed to ascertain the effects of BMI (obese and nonobese), background diseases, lab tests, and nutritional risk on the likelihood that patients discharge from the hospital (Table 4). The odds of discharge from the hospital were 1.20 times greater for patients with higher SF ratio. Increasing SOFA score (3th day) and D-dimer were associated with decreasing odds of survival, and also, the history of BMI > 30, higher PNI score, and NRS risk of nutrition were associated with a reduction in the odds of survival. The comparison of the length of ICU stay in normal and malnourished patients did not show any significant differences (Table 5).

The results of the Pearson correlation test did not show a significant correlation between nutritional tools and length of ICU stay (*P* > 0.05). Also, the length of stay was not significantly different according to the nutrition status (*P* > 0.05).

**Figure 3:**
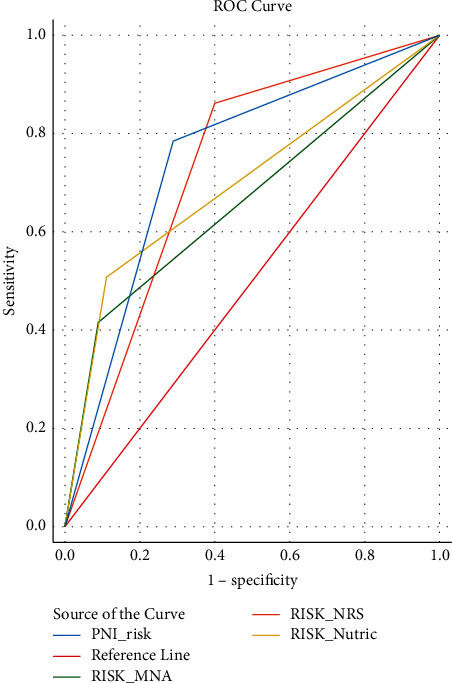
ROC curve for mortality.

The performance of the model was evaluated to predict mortality and mechanical ventilation (MV) using a ROC curve (Tables 6 and 7). The results of ROC curve analysis showed that among the four nutritional tools, PNI (AUC = 0.74, 95% CI (0.651–0.844)) and NRS-2002 (AUC = 0.73, 95% CI (0.630–0.831)) had the highest accuracy in predicting mortality (Figure 3).

**Figure 4:**
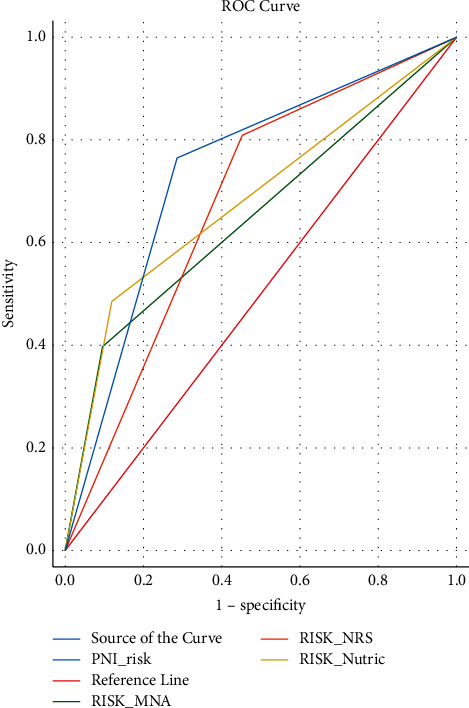
ROC curve for MV.

For the prediction need of mechanical ventilation (Table 6), PNI (AUC = 0.73, 95% CI (0.641–0.838)) and mNUTRIC (AUC = 0.68, 95% CI (0.583–0.783)) had the most capable accuracy (Figure 4).

## 4. Discussion

Among our relatively homogenous sample of COVID-19 patients admitted to the ICU, the prevalence of comorbidities was well matched with the reports of previous studies [2]. Hypertension, followed by diabetes, and cardiovascular diseases were the most commonly observed comorbidities among the participants of this study. As described in the section Methods and Materials, using different nutritional assessment tools (MNA-SF, NRS-2002, mNUTRIC, and PNI), the prevalence of malnourished patients was calculated according to each score's cutoff. Silva et al. reported the rate of malnourished cases as a total of 27.5% among their COVID-19 patients [5]. Our data showed a range of nutritional risk distribution from 28.2% up to 67.2% of cases, with the lowest and highest rates being assessed using MNA and NRS scores, respectively.

For the aim of this study, the associations between nutritional risk and comorbidities are of great importance, as comorbidities increase the risk of malnutrition in patients, which can affect treatment outcomes. The cost of health services and hospitalization periods is reported to be higher in malnourished cancer patients [19]. CKD and malignancy were two comorbidities that were associated with nutritional risk assessed by the mNUTRIC score, and HTN and DM were associated with malnutrition which was evaluated using the MNA-SF score. There are many ways in which the patients' comorbidities could aggravate the nutritional profile. The patient would easily be exposed to malnutrition due to malabsorption and metabolic disorders caused by their underlying disease. A lack of appetite resulted from malignancy. Cachexia induced by cancer treatments like chemotherapy and radiotherapy can increase the risk of malnutrition in these patients.

Interestingly, the higher BMI was associated with a greater chance of developing malnutrition during hospitalization. This finding is in concordance with the former studies; a higher BMI and obesity were shown to be associated with ARDS, mechanical ventilation, and increased mortality [6]. Higher BMI and obesity have been reported to be negative prognostic factors for patient outcome not only in COVID-19 but also in many other pathological situations [20, 21]. Previously, obesity seemed to be a protective factor in cardiovascular diseases, which was referred to as the obesity paradox [22]. It was due to the fact that obese patients had high body fat and also muscle mass, which can play a protective role in cardiovascular disease. If obese patients had sarcopenia (loss of muscle mass), the risk of a poor outcome would be greater. Sarco-obesity, as defined by muscular strength rather than muscle mass, is related to an increased risk of cardiovascular diseases, according to Janssen and Stephen [23]. It is suggested that strength, rather than muscular mass, may be more significant for cardiovascular disease prevention in the elderly. Also, the findings do not suggest that being fat would have increased the survival of nonobese critically ill individuals [24]. However, such a phenomenon could not be found in COVID-19 patients. The exacerbating effects of obesity and higher BMI in critically ill COVID-19 patients could be mainly explained by the altered immune response and malnutrition resulting from pathological changes due to obesity [25].

In this study, APACHE II and SOFA scores were significantly higher in nonsurvival patients as Zhang et al. [11] had presented in their study. As in previous studies, our findings showed that BMI was higher in nonsurvival patients than in survivors and that obesity can increase the need for mechanical ventilation and the likelihood of discharge in ICU patients [6–8]. However, serum albumin was significantly higher in survival patients, and it seems that higher serum albumin is related to better outcomes and lower serum albumin can lead to more severe disease and complications [26]. Albumin is a negative-phase reactant protein, and its association with survival does not reflect malnutrition necessarily. Lower serum albumin can be considered as a severity index in critically ill patients.

We mentioned that expired patients presented with significantly higher WBC, neutrophil count, N/L ratio, and lower lymphocyte count. Evaluating inflammatory biomarkers showed us significantly increased levels of CRP, LDH, and ferritin (except in ESR) in expired patients. These findings were aligned with Michael et al.'s [27] study results. It should be noted that in this study, the serum ESR was elevated in majority of expired patients, but the difference was not statistically significant, and the results of a systematic review [28] revealed that regardless of illness severity or the presence of comorbidities, ESR and CRP values were elevated. McNeil et al. [29] suggested that the higher level of D-dimer and CRP is associated with poor prognosis of patients after adjusting BMI that is consistent with the results of our study. Higher BMI, D-dimer, and CRP significantly increase the risk of hospital death. In contrast with Fawad Rahim et al.' [30] findings, we demonstrated that there is no any significant association between mortality and length of ICU stay days, but a study performed by Mahendra et al. [31] in India concluded that there is a significant difference in the length of ICU stay days of survived COVID-19 patients with severe pneumonia and expired ones.

Our finding about the significant difference in SF ratio between survived and nonsurvived patients was similar to Mahendra et al.'s study [31]. It was found that higher mNUTRIC and NRS-2002 and lower PNI and MNA-SF scores are associated with an increased risk of mortality. Nutritional risk, according to all tools, can independently predict the need for mechanical ventilation and mortality. This has been proven in various previous studies [11, 12].

The results of logistic regression showed that a higher PNI score and SF ratio increase the odds of survival, but increasing D-dimer, SOFA score (day 3), NRS, and BMI (more than 30) intensify the risk of death. This finding is aligned with the previous study [12] that predicts the mortality of patients by inflammatory biomarkers and nutrition tools.

In this study, we determined the length of hospital stay in the intensive care unit of patients using multiple linear regression modeling. The history of HTN, RDW-CV, and N/L ratio could independently predict the length of ICU stay of COVID-19 patients. Previous studies [32–34] revealed that malnutrition is associated with longer hospital stay. However, our results suggested predictors of length of ICU stay, and we did not find any significant difference in length of ICU stay between normal and malnourished patients.

The results of ROC curve analysis showed that, in terms of mortality prediction, NRS-2002 and PNI scores are highly sensitive. Furthermore, for the prediction of mechanical ventilation, PNI and mNUTRIC scores are more powerful.

### 4.1. Limitations

This study has two limitations. First, we failed to assess muscle and fat mass, which can reflect a history of malnutrition and sarcopenia. Second, we failed to determine the controlling nutritional status score (CONUT) and some other important criteria in nutritional evaluation, such as the panel of lipoproteins and cholesterol. It is suggested to consider all the mentioned factors in future studies. Finally, this was a single-center study that covered COVID-19 patients during the 3rd and 4th COVID-19 waves in Iran. So, different types of patients' ethnicities and also various SARS-CoV-2 variants may have different pathophysiology and, accordingly, different effects on the nutritional status of COVID-19 patients.

We have excluded patients aged more than 80 years. This is due to the fact that patients with age more than 80 may have different comorbidities that can influence their nutritional status. Also, according to the APACHE score, they are more likely to have a poor outcome when admitted to ICU. Also, intubated patients and/or those with multiorgan failures are highly at risk of malnutrition. They received different types of medication that influenced their nutritional status. So, intubated status of organ failure seemed to be a confounder; therefore, we have excluded these patients.

## 5. Conclusion

In the critical care setting of COVID-19, patients' malnutrition is prevalent. CKD, DM, HTN, and cancer patients are more likely to be malnourished at the time of ICU admission and should be screened for nutritional needs and treatments. COVID-19 patients' mortality rates are highly sensitive to NRS-2002 and PNI scores. Also, PNI and mNUTRIC scores are the most sensitive tools to predict the need for mechanical ventilation. Malnutrition (nutritional risk) is associated with an increased risk of need for mechanical ventilation and in-hospital mortality. Higher BMI, ferritin, N/L ratio, and lower albumin and SF ratio are the risk factors for hospital death. It seems that the history of HTN, RDW-CV, and N/L ratio could independently predict the length of ICU stay of COVID-19 patients.

## Figures and Tables

**Table 1:** Laboratory test comparison between survival and nonsurvival patients.

Variable	Mean ± standard deviation		*P*
	Expired	Discharged	
BMI (kg/m^2^)	28 ± 6	24 ± 3	<0.001
SpO_2_ (%)	85 ± 5	90 ± 4	<0.001
APACHE II	18 ± 5	14 ± 3	<0.001
SOFA1	6 ± 3	4 ± 2	<0.001
SOFA2	7 ± 3	4 ± 2	<0.001
SOFA3	8 ± 3	4 ± 2	<0.001
NRS-2002	4 ± 1	2 ± 1	<0.001
MNA-SF	11 ± 1	12 ± 1	<0.001
mNUTRIC	4 ± 2	2 ± 1	<0.001
PNI	31 ± 6	40 ± 8	<0.001
CRP (mg/dl)	101 ± 53	60 ± 57	<0.001
ESR (mm/h)	53 ± 23	45 ± 20	0.051
LDH (U/L)	969 ± 397	776 ± 366	0.006
Ferritin (ng/dl)	625 ± 335	308 ± 253	<0.001
Albumin (g/dl)	2 ± 0.5	3 ± 0.6	<0.001
WBC (10^9^/L)	12072 ± 5187	9284 ± 5117	0.006
Neutrophil count	10776 ± 4780	7510 ± 4469	<0.001
Lymphocyte count	775 ± 713	1210 ± 1018	0.01
N/L ratio	23 ± 18	10 ± 11	<0.001
SF ratio	107 ± 7	113 ± 5	<0.001
Length of stay (days)	9 ± 5	8 ± 4	0.29
RDW^*∗*^-CV^*∗∗*^ (%)	14.4 ± 1.8	14.7 ± 1.3	0.33
RDW-SD^*∗∗∗*^ (fL)	47.7 ± 6.6	47.2 ± 6.1	0.72

^*∗*^RDW, red cell distribution width; ^*∗∗*^CV, coefficient of variation; ^*∗∗∗*^SD, standard deviation.

**Table 2:** Cross-tabulation of BMI categories and patients' outcomes.

	Outcome		Total	*P* value
	Expired	Discharged		
Underweight	2 (40.0%)	3 (60.0%)	5	0.001
Normal weight	19 (45.2%)	23 (54.8%)	42	
Overweight	12 (44.4%)	15 (55.6%)	27	
Obesity class 1	19 (86.4%)	3 (13.6%)	22	
Obesity class 2	10 (90.9%)	1 (9.1%)	11	
Obesity class 3	3 (100%)	0 (0%)	3	

**Table 3:** Cross-tabulation of nutritional risk, hospital discharge, and need for mechanical ventilation.

Nutritional risk	Discharge		Mechanical ventilation	
	OR (95% CI)	*P*	OR (95% CI)	*P*
mNUTRIC	0.121 (0.042–0.346)	<0.001	6.97 (2.44–19.89)	<0.001
NRS-2002	0.107 (0.043–0.270)	<0.001	5.121 (2.17–12.06)	<0.001
MNA-SF	0.137 (0.044–0.429)	<0.001	6.25 (2–19.54)	0.001
PNI	0.112 (0.046–0.267)	<0.001	8.12 (3.39–19.45)	<0.001

**Table 4:** Logistic regression for predicting discharge from hospital.

		Sig.	Odds ratio	CI 95%	
Discharge from the hospital	BMI category	0.014	0.097	0.015	0.63
	SOFA3	0.005	0.611	0.432	0.864
	PNI score	0.013	1.162	1.032	1.389
	RISK NRS	0.001	0.039	0.006	0.275
	D-dimer	0.006	0.997	0.995	0.999
	SF ratio	0.015	1.20	1.036	1.310
	Overall model	0.023	0.000		

**Table 5:** Length of ICU stay in normal and malnourished patients.

Malnutrition		Mean ± standard (IQR)	*P*
NRS	Yes	8.70 ± 5.20 (7)	0.96
	No	8.75 ± 5.30 (4.5)	
MNA	Yes	9.67 ± 5.24 (8)	0.23
	No	8.34 ± 5.21 (5)	
mNUTRIC	Yes	7.63 ± 4.43 (6)	0.11
	No	9.29 ± 5.53 (7)	
PNI	Yes	9.20 ± 5.58 (6)	0.25
	No	8.04 ± 4.69 (6)	

**Table 6:** ROC curves in predicting mortality in nutrition tools.

Mortality (in-hospital death)			
Test result variable(s)^*∗*^	Area	*P*	95% confidence interval
NRS	0.731	<0.001	0.630–0.831
MNA	0.663	0.001	0.563–0.764
mNUTRIC	0.698	<0.001	0.600–0.796
PNI	0.748	<0.001	0.651–0.844

**Table 7:** ROC curves in predicting mechanical ventilation in nutrition tools.

Mechanical ventilation			
	Area	*P*	95% confidence interval
NRS	0.678	0.001	0.571–0.785
MNA	0.651	0.004	0.549–0.753
mNUTRIC	0.683	<0.001	0.583–0.783
PNI	0.739	<0.001	0.641–0.838

^*∗*^Nutritional risk according to the different tools.

## Data Availability

The data used to support the findings of this study are included within the article.
